# Thermophysically Guided Design of Spray-Dried *Lactiplantibacillus plantarum* Microcapsules with Improved Thermal Stability

**DOI:** 10.3390/foods15203749

**Published:** 2026-10-21

**Authors:** Ana Maria Naranjo Duran, Julian Quintero-Quiroz, Eimy Sandrith Jimenez Rodriguez, Karen Yulieth Vargas Gonzalez, Néstor Giraldo, Bernadette Klotz

**Affiliations:** 1 College of Food and Pharmaceutical Sciences, CES University, Calle 10 # 22-04, Medellin 050018, Colombia; anaranjod@ces.edu.co (A.M.N.D.); jimenez.eimy@uces.edu.co (E.S.J.R.); karenyuliethvago@ufps.edu.co (K.Y.V.G.); 2 Alpina Productos Alimenticios S.A.S. BIC, Km 3 vía Briceño-Sopó, Edificio Administrativo Alpina, Sopó 251001, Colombia; nestor.giraldo@alpina.com (N.G.); bernadette.klotz@alpina.com (B.K.)

**Keywords:** apparent activation energy, thermoprotectant, gastrointestinal survival, Kissinger method

## Abstract

Spray drying and gastrointestinal transit can cause substantial losses in probiotic viability, limiting effective delivery in food applications. This study developed a thermophysically guided framework for the rational design of *Lactiplantibacillus plantarum* microcapsules in three sequential stages. First, Kissinger kinetic analysis of eleven biopolymer candidates (*E*_a_: 95.4–198.4 kJ/mol) combined with pH-dependent solubility profiling provided an empirical screening criterion for the preselection of coating materials; the activation energies are apparent values associated with a composite endothermic event and the glass transition temperature was not measured in this work. Second, a factorial design identified whey protein concentrate (WPC)–gum arabic as the most suitable wall system, yielding a post-drying survival of 97.76 ± 0.75% and showing a significant protein–polysaccharide interaction (*p* < 0.0001). Third, a D-optimal mixture design optimized a four-component thermoprotectant blend composed of trehalose (29.7%), glycerol (25.7%), fructooligosaccharides (19.8%), and inulin (24.8%). The fitted model explained 91.9% of the response variability (R^2^ = 0.919), and the optimized blend limited viability loss during thermal exposure at 92 °C for 3–8 min, consistent with complementary protective effects involving vitrification and membrane stabilization. The optimized system yielded 2.4 × 10^11^ CFU/g after spray drying, a water activity of 0.23, and a Carr Index of 18.5%. Under simulated gastrointestinal digestion (INFOGEST 2.0), thermoprotectant-loaded microcapsules retained 1.5 × 10^7^ CFU/mL of digesta after the gastric phase and delivered 1.2 × 10^8^ CFU/mL of digesta to the intestinal phase, four to five orders of magnitude above unprotected microcapsules (1.0 × 10^3^ CFU/mL of digesta). Overall, these results support a transferable formulation strategy that combines thermophysical screening with experimental design to develop probiotic ingredients with improved thermal stability for food applications.

## 1. Introduction

The modulation of the gut microbiota through probiotic supplementation is a well-established therapeutic strategy, with demonstrated applications in irritable bowel syndrome, inflammatory bowel disease, and the attenuation of systemic inflammatory biomarkers [1,2]. Despite this evidence, the large-scale commercialization of probiotic products is constrained by a critical technological limitation that markedly compromises their clinical effectiveness. Viability losses occurring during industrial processing, storage in the absence of a continuous cold chain, and gastrointestinal transit may collectively reach 3–5 logarithmic cycles, resulting in a decline from initial concentrations of approximately 10^9^ CFU/g to subtherapeutic levels (below 10^6^ CFU/g). This limitation impacts both manufacturers and consumers; from an industrial perspective, it leads to reduced product efficacy, shorter shelf life, and increased costs associated with over-dosification strategies, while from the consumer standpoint it translates into products that fail to deliver meaningful health benefits [3].

Spray drying, the industrial standard method for probiotic dehydration due to its scalability and cost-effectiveness, constitutes the first major source of cellular stress in probiotic manufacturing. Inlet air temperatures typically ranging from 140 to 180 °C generate severe thermal gradients that expose microbial cells to protein denaturation, lipid peroxidation of cell membranes, and rapid osmotic dehydration [4]. Recent studies using *Lactiplantibacillus plantarum* have shown that when outlet temperatures exceed 70 °C, cell survival decreases markedly, with post-drying viabilities ranging from 75.70% to 99.73%, depending primarily on the coating material employed [5,6]. The choice of the encapsulating agent critically determines the extent of thermal protection afforded to probiotic cells during both the constant-rate and falling-rate drying periods [7].

Beyond processing, the contemporary food industry increasingly incorporates probiotics into products that undergo thermal treatments, including pasteurized beverages (72 °C for 15 to 90 s), baked goods, and reconstituted or UHT-treated foods, among other matrices that impose combined heat and oxidative stresses on microbial cells. In such systems, probiotics experience an acute thermal insult during processing followed by prolonged exposure to ambient storage temperatures. Studies with encapsulated *Lactiplantibacillus plantarum* show that free cells exhibit substantial inactivation at temperatures above 50 °C, whereas alginate–soy protein microcapsules can retain 10^6^–10^8^ CFU/mL after treatment at 72 °C for 90 s [8]. However, in complex acidic matrices such as fruit juices (pH 3.5 to 4.5), rapid dissolution of hydrophilic polysaccharides may cause premature capsule solubilization and early probiotic release, compromising targeted intestinal delivery [9].

To address these challenges, coating material selection must be guided by quantifiable thermophysical criteria rather than empirical trial-and-error approaches. Among them, the glass transition temperature (*T*_g_) is a key determinant of amorphous matrix stability. Materials with *T*_g_ values sufficiently above the storage or processing temperature provide superior protection by maintaining the encapsulant in a glassy, low-mobility state, whereas systems operating below their *T*_g_ are prone to structural collapse, crystallization, and accelerated oxygen diffusion [10,11]. For instance, powders containing 10% gum arabic exhibit higher *T*_g_ than maltodextrin, trehalose, or their binary blends, which explains the superior survival of *L. plantarum* during isothermal heating at 90 °C [12]. Trehalose, with an anhydrous *T*_g_ of approximately 115 °C and molecular relaxation activation energies between 175 and 400 kJ/mol, is an effective thermoprotectant at 10 to 50% (*w*/*v*) in industrial processing [13,14]. Protein–polysaccharide matrices further enhance protection by combining film-forming proteins (whey protein isolate, soy protein, calcium caseinate, and food-grade collagen) with polysaccharides that contribute interfacial stability, enzymatic resistance, and structural flexibility, such as octenyl succinic anhydride (OSA)-modified cassava starch, gum arabic, and sodium alginate [15,16]. During spray drying, proteins undergo rapid surface denaturation at the air–droplet interface, forming a continuous viscoelastic film that limits heat and mass transfer to the particle interior, while the polysaccharide fraction raises the effective *T*_g_ of the consolidated matrix and contributes to hydrogen-bond-mediated water immobilization [7]. In parallel, low-molecular-weight thermoprotectants including trehalose, fructooligosaccharides, inulin, and glycerol can increase the effective *T*_g_, replace structural water at phospholipid headgroup interfaces, and provide osmotic stabilization of the intracellular milieu, raising survival from negligible levels without inulin to 60 to 70% at 40% inulin and preserving up to 98% viability after two months of storage [4,17].

Despite these advances in probiotic encapsulation, the selection of coating materials and thermoprotectants remains largely empirical, with limited integration of quantitative thermophysical criteria into the formulation design process. Recent reviews on probiotic encapsulation and stability highlight that most industrial formulations still rely on empirical combinations of wall materials and protectants, with only sporadic use of glass transition or kinetic parameters as explicit design criteria [18]. In particular, no systematic framework has been reported that combines thermal kinetic analysis of candidate materials with statistically designed screening of wall system combinations and thermoprotectant mixtures within a single, integrated experimental strategy. This work addresses this gap through a systematic three-stage experimental strategy for *Lactiplantibacillus plantarum* microencapsulation. First, Kissinger-based kinetic analysis and pH-dependent solubility profiling of eleven biopolymer candidates were used to establish quantitative preselection criteria. Second, a 3 × 2 factorial design evaluated binary protein–polysaccharide combinations to identify the wall system maximizing both post-drying survival and viable count. Third, a D-optimal mixture design optimized a quaternary thermoprotectant formulation to enhance probiotic survival under thermal conditions representative of food pasteurization, using cell survival after treatment at 92 ± 2 °C for 2 min as the design response; the formulations generated by numerical optimization were subsequently validated at longer exposure times (92 °C for 3, 5, and 8 min). The resulting optimized system was characterized for physicochemical properties and evaluated for gastrointestinal resistance using the standardized INFOGEST 2.0 protocol. We hypothesized that integrating thermophysical parameters (thermal transition energies and pH-dependent solubility) into the material selection process would yield encapsulation systems with superior thermal and gastrointestinal performance compared to empirically selected formulations. The resulting microcapsules are intended for use as a dry probiotic ingredient in thermally processed food products, namely pasteurized dairy and plant-based beverages, fermented dairy products, fruit-based drinks, and dry-blended or baked matrices such as powdered beverage mixes, cereal bars, and bakery fillings, where the powder must withstand a short heat treatment during processing and subsequent gastric transit before releasing viable cells in the intestine.

## 2. Materials and Methods

### 2.1. Materials

Gum arabic (95%), OSA-modified cassava starch (95.3%), sodium alginate, guar gum, xanthan gum, and microcrystalline cellulose were used as polysaccharide coating materials. The proteins evaluated included sodium caseinate, calcium caseinate, whey protein concentrate (WPC, ≥80%), soy protein isolate (SPI, ≥90%), and food-grade collagen. All materials were food-grade and sourced from local commercial suppliers in Medellín, Colombia. The thermoprotectants trehalose, glycerol, inulin, and fructooligosaccharides (FOS) were obtained from the same commercial sources. De Man, Rogosa and Sharpe (MRS) culture medium, bile salts, pepsin, pancreatin, and other analytical-grade reagents (ACS grade) were purchased from Merck KGaA (Darmstadt, Germany) or equivalent local distributors. All water used throughout the study was Type I ultrapure water.

### 2.2. Bacterial Cultures

The probiotic strain *Lactiplantibacillus plantarum (ATCC 8014)* was obtained from the culture collection of the Corporación Centro de la Ciencia y la Investigación Farmacéutica (CECIF), Colombia. The strain was preserved as glycerol stocks (30%, *v*/*v*) at −80 °C and subcultured twice in MRS broth at 37 °C and 100 rpm for 18 h prior to each experiment.

### 2.3. Thermal Characterization of Coating Materials

#### 2.3.1. Differential Scanning Calorimetry (DSC)

The thermal properties of the candidate coating materials were evaluated using a DSC3+ instrument (Mettler Toledo, Schwerzenbach, Switzerland). Samples (5–10 mg) were hermetically sealed in 40 μL aluminum crucibles and analyzed under a nitrogen atmosphere (30 mL/min). Thermograms were recorded from 25 °C to 200 °C at heating rates of 5, 10, and 20 °C/min. The following materials were characterized by DSC: gum arabic, sodium caseinate, guar gum, calcium caseinate, sodium alginate, xanthan gum, microcrystalline cellulose, soy protein isolate (SPI), whey protein concentrate (WPC), food-grade collagen, and OSA-modified cassava starch. Thermal transition parameters, including the onset temperature of the endothermic transition (*T*_onset_), endset temperature, enthalpy of the endothermic transition (ΔH), and peak width, were determined using STARe Evaluation Software (version 18.0, Mettler Toledo). An empty sealed crucible was used as a reference in all measurements. Each material was analyzed in triplicate.

#### 2.3.2. Activation Energy Calculation

The activation energy (*E*_a_) associated with the endothermic thermal transitions of the coating materials was calculated using the Kissinger method [19]. This approach is based on the relationship between the heating rate (*β*) and the temperature at which the transition rate reaches its maximum (*Tp*), as described by the following equation:

(1) $$\ln\left(\frac{\beta }{T_{p}^{2}}\right)=\ln\left(\frac{\mathrm{AR}}{E_{a}}\right)-\frac{E_{a}}{RT_{p}}$$

where

*β* is the heating rate (°C/min);

*Tp* is the peak transition temperature (K);

*A* is the pre-exponential factor (min^−1^);

*R* is the universal gas constant (8.314 J/mol·K);

*E_a_* is the activation energy (kJ/mol).

*E_a_* was determined from the slope of the linear plot of ln(*β*/*Tp*^2^) versus 1/*Tp*, where the slope equals −*E_a_*/*R* [20]. Peak temperatures were obtained from DSC thermograms recorded at 5, 10, and 20 °C/min, and the coefficient of determination (R^2^) was used to assess the goodness of fit of the linear regression [21]. The *E_a_* values obtained reflect the energy barrier for the composite process of bound water desorption and macromolecular chain relaxation, which governs the thermal stability of the amorphous matrix during drying and storage rather than covalent bond degradation of the polymer backbone [10]. This interpretation is consistent with previous work on amorphous carbohydrate mixtures, where the activation energy of endothermic transitions has been used as a proxy for structural stability and resistance to collapse under processing and storage conditions [22].

#### 2.3.3. Solubility of Coating Materials and Thermoprotectants

The solubility of the five coating materials selected for the encapsulation factorial design—whey protein concentrate (WPC), soy protein isolate (SPI), calcium caseinate, gum arabic, and OSA-modified cassava starch—and for the thermoprotectants—trehalose, glycerol, inulin, and fructooligosaccharides (FOS)—was determined at two pH values: pH 4.0 and pH 8.0. These two values were selected because they represent one acidic and one alkaline condition bracketing the pH range relevant to the intended applications and to gastrointestinal transit: pH 4.0 approximates acidic food matrices such as fermented dairy products and fruit beverages, whereas pH 8.0 represents the alkaline conditions encountered in the intestinal environment. A 1% (*w*/*v*) suspension of each material was prepared in 0.1 M phosphate buffer previously adjusted to pH 4.0 or pH 8.0 with 1 M HCl or 1 M NaOH as appropriate. Suspensions were incubated at 37 °C for 60 min under continuous agitation at 150 rpm and then centrifuged at 3000× *g* for 10 min. The supernatant was collected and dried at 105 °C to constant weight. Solubility was expressed as the percentage of dissolved solids relative to total solids initially dispersed [23]. All measurements were performed in triplicate.

### 2.4. Experimental Design

#### 2.4.1. Factorial Screening of Protein–Polysaccharide Combinations

A completely randomized 3 × 2 categorical factorial design was employed to evaluate protein–polysaccharide wall-material combinations for the spray drying microencapsulation of *L. plantarum*. The factors evaluated were protein type (whey protein concentrate [WPC], soy protein isolate [SPI], and calcium caseinate), and polysaccharide type (gum arabic and OSA-modified cassava starch), combined at a 1:1 (*w*/*w*) protein-to-polysaccharide ratio to reach a total solids concentration of 10% (*w*/*v*), i.e., 5% protein and 5% polysaccharide [24]. The design comprised six treatment combinations, each performed in triplicate (*n* = 3), yielding a total of 18 experimental units. Response variables were post-drying viable count (CFU/g) and post-drying survival (%). All formulations were processed by spray drying as described in Section 2.5.

#### 2.4.2. Mixture Design for Thermoprotectants

A D-optimal mixture design was employed to optimize thermoprotectant blends using Design-Expert software version 13.0 (Stat-Ease Inc., Minneapolis, MN, USA). The components evaluated were trehalose (X_1_: 0–100%), glycerol (X_2_: 0–100%), inulin (X_3_: 0–100%), and fructooligosaccharides (FOS; X_4_: 0–100%), with the fundamental mixture constraint X_1_ + X_2_ + X_3_ + X_4_ = 1.0, equivalent to a total thermoprotectant concentration of 2.5% (*w*/*v*) in the encapsulation solution. The experimental design comprised 20 runs, including pure-component vertices, binary, ternary, and quaternary mixtures, as well as centroid points to ensure adequate coverage of the mixture space. Post-thermal treatment cell survival (%) was the only response variable evaluated in this design. Thermal treatment of the design runs was carried out in a thermostatic water bath at 92 ± 2 °C for 2 min, under the conditions described in Section 2.6.2, yielding a single response value per run. The additional exposure times of 3, 5, and 8 min were not part of the mixture design and were applied only to the five candidate formulations generated by numerical optimization during the subsequent validation stage (Section 3.3) [25]. The challenge temperature of 92 °C was selected to represent a severe thermal condition relevant to thermally processed dairy and beverage systems [26].

Model validation was performed based on ANOVA, R^2^, adjusted R^2^, predicted R^2^, and lack-of-fit tests. Numerical optimization was conducted using a desirability function approach to maximize the survival of *Lactiplantibacillus plantarum* during thermal processing.

### 2.5. Microencapsulation by Spray Drying

#### 2.5.1. Inoculum Preparation

*Lactiplantibacillus plantarum* cultures were prepared by inoculating 1% (*v*/*v*) of an activated culture into 500 mL of MRS broth and incubating at 37 °C and 100 rpm for 18 h. It was confirmed that the cultures had reached the early stationary phase (~10^9^ CFU/mL) before harvest. Cells were harvested by centrifugation at 8000× *g* for 10 min at 4 °C, washed twice with sterile phosphate-buffered saline solution (PBS, pH 7.2, 0.85% NaCl), and resuspended in the encapsulation solution [27].

#### 2.5.2. Preparation of the Feed Solution

The feed solution was prepared by dissolving the selected coating materials and thermoprotectants in sterile ultrapure water at room temperature (22 ± 2 °C) to achieve a total solids content of 10% (*w*/*v*), with protein and polysaccharide combined at a 1:1 (*w*/*w*) ratio (5% + 5%), under continuous stirring until a homogeneous dispersion was obtained. The bacterial suspension was then added to the feed solution; the 10% (*w*/*v*) figure refers to the wall materials only and does not include the added bacterial biomass. In the thermoprotectant-loaded formulations, the optimized thermoprotectant blend was added at 2.5% (*w*/*v*) in addition to the wall system (Section 2.4.2), so that the feed delivered to the dryer contained 12.5% (*w*/*v*) total solids, whereas the unprotected control contained 10% (*w*/*v*). The mixture was homogenized using a high-shear homogenizer at 5000 rpm for 1.5 min to ensure uniform dispersion of all components prior to atomization [28]. Preliminary standardization assays in which viable counts (CFU/mL) were measured before and after this step confirmed that high-shear homogenization did not reduce the viability of the bacterial culture prior to drying. The feed solution was maintained at 4 °C for no longer than 30 min before spray drying to minimize cell loss prior to processing.

#### 2.5.3. Spray Drying Process

Spray drying was carried out using a Büchi Mini Spray Dryer B-290 (BÜCHI Labortechnik AG, Flawil, Switzerland) equipped with a standard two-fluid nozzle atomizer (nozzle tip diameter: 0.7 mm). The operating conditions were as follows: inlet air temperature of 110 ± 2 °C, feed flow rate 15% of maximum speed of the peristaltic pump, atomization air flow rate set at 35 mm on the rotameter (≈414 L/h, according to the manufacturer’s conversion table), and aspirator rate of 100%. The resulting outlet air temperature was 65 ± 2 °C. The resulting powder was collected from the high-performance cyclone separator, weighed to determine process yield (%), and immediately stored at 4 °C in aluminum-laminated, heat-sealed bags until further analysis (within 48 h of production). Process yield was calculated as the ratio of collected powder mass to total solids in the feed solution, expressed as a percentage [23].

### 2.6. Microcapsule Characterization

#### 2.6.1. Probiotic Survival After Spray Drying

Probiotic viability after spray drying was determined by the plate count method. Briefly, 100 mg of spray-dried powder was rehydrated in 0.9 mL of phosphate buffer (pH 8.0), vigorously mixed for 5 min, and serially diluted. Appropriate dilutions were plated on MRS agar and incubated at 37 °C for 48 h under anaerobic conditions. Viable counts were determined both in the feed suspension before drying and in the resulting powder, and were converted to a dry-solids basis using the moisture content of each sample (Section 2.6.3), so that both terms of the ratio are expressed as CFU per gram of dry solids and are therefore directly comparable. Post-drying survival was calculated as the ratio of the viable count recovered in the powder to that of the corresponding feed suspension and, in parallel, as the reduction in log cycles between the two counts, as follows:

(2) S(%)=N1N0×100, Δlog N=logN0−logN1

where N_1_ is the viable count of the powder after drying and N_0_ is the viable count of the corresponding feed suspension before drying, both expressed as CFU per gram of dry solids [23,29]. Referring both counts to the same dry-solids basis ensures that S represents the fraction of viable cells recovered after drying and is not affected by the dilution of the cells in the dry matrix. The reduction in log cycles (Δlog N) is reported in parallel, since it is the conventional metric in the microbiological literature and is less sensitive to small absolute differences at high survival levels. Because each stage of the experimental design was carried out with an independently prepared culture, N_0_ was determined for every run rather than assumed constant across the design. This parameter quantifies the survival of the culture through the drying step and is not an encapsulation efficiency in the strict sense, since the latter requires washing the particle surface to remove non-encapsulated cells and quantifying only the fraction retained within the matrix, a procedure that was not performed in this work. Accordingly, the term survival is used throughout the manuscript, and the scanning electron micrographs are interpreted as qualitative evidence of particle morphology and wall continuity rather than as evidence of the spatial distribution of cells within or on the particles.

#### 2.6.2. Thermal Resistance of Microcapsules

The thermal resistance of the optimized microcapsules was evaluated by suspending 1% (*w*/*v*) of spray-dried powder in sterile ultrapure water (as specified in Section 2.1). The suspension was subjected to a constant temperature of 92 ± 2 °C in a thermostatic water bath for 2 min with continuous gentle agitation. Immediately following heat treatment, samples were transferred to an ice bath for 5 min to halt thermal inactivation and prevent residual heat carry-over effects. The suspension was then collected and *L. plantarum* viability was determined by plate counting on MRS agar as described in Section 2.6.1. Results were expressed as CFU/g of powder and as thermal resistance, defined as the percentage of viable cells surviving the heat treatment relative to the untreated post-drying powder [26,30], as described by the following equation:

(3) $$\mathrm{TR}(\%)=\frac{N_{H}}{N_{D}}\times 100$$

where N_H_ is the viable count after the heat treatment and N_D_ is the viable count of the untreated post-drying powder, both expressed as CFU per gram of dry solids. As in Equation (2), thermal resistance is calculated as an arithmetic ratio of viable counts; because both terms refer to the same powder, they share the same solids basis and the same order of magnitude, and the corresponding reduction in log cycles is reported alongside the percentage values where relevant.

#### 2.6.3. Physicochemical Properties

Moisture content was determined using an infrared moisture analyzer (Aczet 129, Mumbai, India) by heating 2 g of sample at 102 ± 2 °C until constant weight was achieved. Water activity (aw) was measured at 25 °C using a Novasina LabSwift Portable Water Activity Meter (Novasina, Lachen, Switzerland). Particle size distribution was determined by laser diffraction using a Mastersizer 3000 (Malvern Panalytical, Worcestershire, UK) under dry dispersion conditions. Flow properties were evaluated using Carr index and Hausner ratio, calculated from bulk and tapped densities [31].

#### 2.6.4. Scanning Electron Microscopy (SEM)

Microcapsule morphology and surface structure were examined using scanning electron microscopy. Samples were mounted on aluminum stubs and sputter-coated with a gold layer (10–15 nm) using a sputter coater operated at 20 mA for 90 s [28].

Micrographs were acquired at magnifications of 500×, 1000×, 2500×, and 5000×, using an accelerating voltage of 20 kV, to qualitatively assess particle shape, size, surface texture, sphericity, presence of indentations, and overall structural integrity of the microcapsules [24,28].

#### 2.6.5. Evaluation of Gastrointestinal Resistance

The survival of encapsulated and free *L. plantarum* under simulated gastrointestinal conditions was assessed using the INFOGEST 2.0 static protocol [32]. The simulated salivary, gastric and intestinal fluids (SSF, SGF and SIF) were prepared from the electrolyte stock solutions specified in the protocol (KCl, KH_2_PO_4_, NaHCO_3_, NaCl, MgCl_2_·6H_2_O, (NH_4_)_2_CO_3_ and CaCl_2_) at the concentrations given therein, and each digestive phase was carried out at the prescribed 1:1 (*v*/*v*) ratio of simulated fluid to sample, with CaCl_2_ and the enzymes added to the final activities specified by the protocol. Oral phase: 0.5 g of powder was first reconstituted in 4.5 mL of water to give 5 mL of a 10% (*w*/*v*) food matrix, since the protocol requires a fluid food as the starting material; this reconstituted matrix was then mixed with 5 mL of simulated salivary fluid (pH 7.0, α-amylase 75 U/mL), corresponding to the prescribed 1:1 (*v*/*v*) ratio, and incubated at 37 °C for 2 min at 150 rpm. The water therefore serves to reconstitute the dry powder into the food matrix and does not dilute the simulated fluid, so the final electrolyte and enzyme concentrations in each phase are those of the standard protocol. Gastric phase: pH was adjusted to 3.0 with 1 M HCl, pepsin (2000 U/mL) was added, and the mixture was incubated for 120 min. Intestinal phase: pH was adjusted to 7.0 with 1 M NaOH, pancreatin (100 U/mL) and bile salts (10 mM) were added, and incubation continued for 120 min. Viable cell counts were performed at the end of each phase to quantify viable-cell recovery of *L. plantarum* in each gastrointestinal compartment [33].

### 2.7. Statistical Analysis

**Figure 1:**
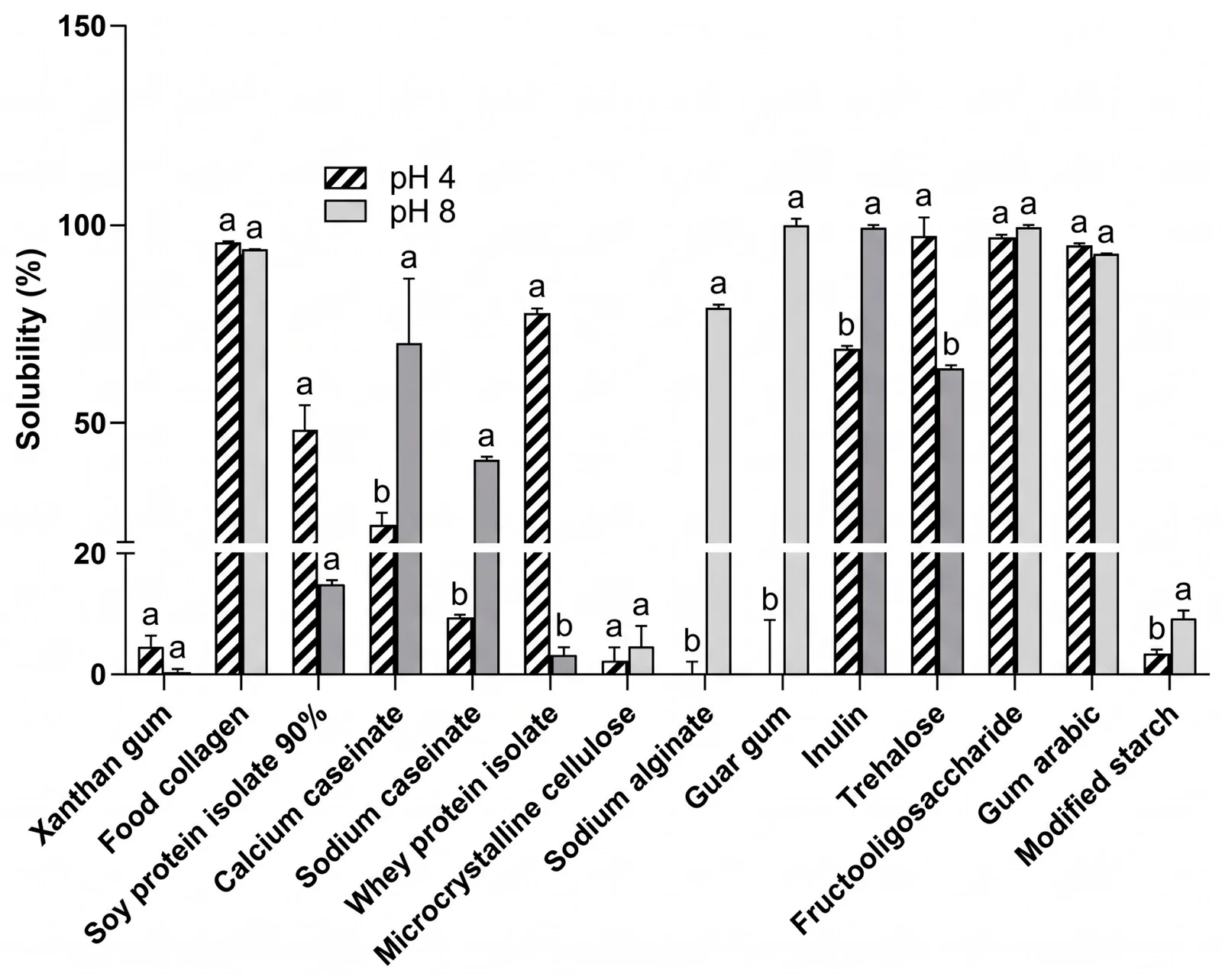
pH-dependent solubility (%) of the five coating materials and four thermoprotectants selected for experimental design stages, determined at pH 4.0 and pH 8.0. Values represent mean ± SD (*n* = 3). Different lowercase letters indicate statistically significant differences between pH conditions within each material (*p* < 0.05; Tukey’s HSD test).

**Figure 2:**
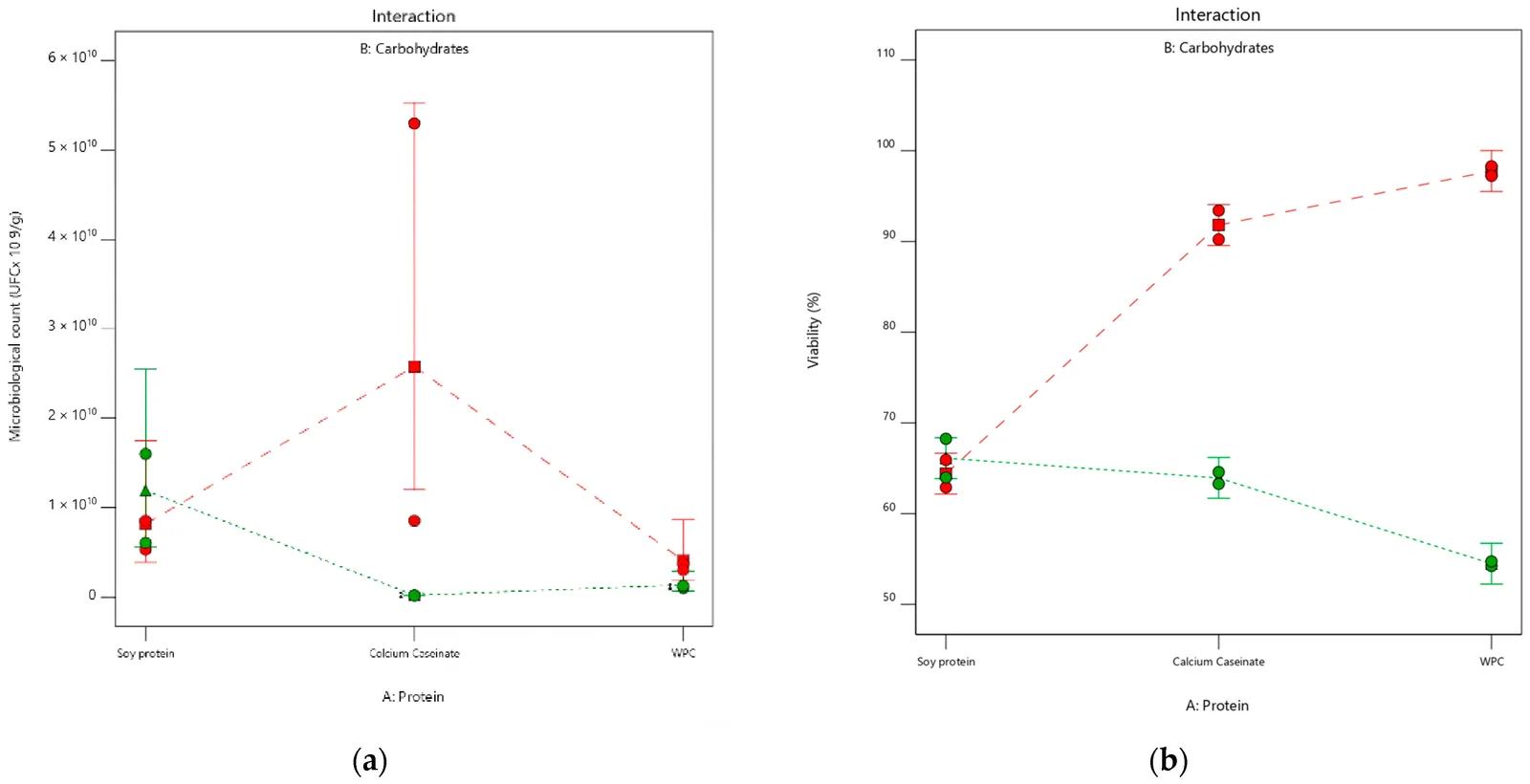
Mean response values from the 3 × 2 factorial design for *L. plantarum* microencapsulation. (**a**) Post-drying viable count (CFU/g) as a function of protein type (Factor A: WPC, SPI, calcium caseinate) and polysaccharide type (Factor B: gum arabic, OSA-modified cassava starch); (**b**) Survival rate (%) as a function of the same factorial combinations. In both panels, the red line corresponds to gum arabic and the green line to OSA-modified cassava starch. Error bars represent standard deviation (*n* = 3).

All experiments were conducted in triplicate, and results were expressed as mean ± standard deviation. Statistical analyses were performed using STATGRAPHICS Centurion 19 software. Comparisons involving three or more means—the DSC parameters of the coating materials at each heating rate (Table 1), the pH-dependent solubility data (Figure 1) and the six treatment combinations of the factorial design (Table 2 and Figure 2; viable counts were log_10_-transformed prior to analysis)—were analyzed by analysis of variance (ANOVA) followed by Tukey’s HSD post hoc test (*p* < 0.05). The 3 × 2 factorial design was analyzed by two-way ANOVA including the protein × polysaccharide interaction term. Comparisons between the two optimized formulations, with and without thermoprotectant, involve only two groups and were therefore assessed by a two-sample Student’s *t*-test (*p* < 0.05). For the in vitro digestion assay, viable counts were log_10_-transformed; within each digestive phase, the three treatments were compared by one-way ANOVA followed by Tukey’s HSD test (*p* < 0.05), and gastric and intestinal counts of each treatment, obtained from the same digestion run (*n* = 3 independent runs), were compared by a paired Student’s *t*-test (*p* < 0.05). For the D-optimal mixture design, the significance of individual and interaction terms was evaluated within the ANOVA of the fitted model rather than by pairwise post hoc comparison, because the runs are design points of the mixture space and not independent treatment groups. Activation energies obtained by the Kissinger method were derived by linear regression and are reported with their coefficients of determination. For experimental design analyses, Design-Expert software version 13 was used. A special cubic Scheffé polynomial model was fitted to the mixture-design data and validated based on residual analysis, coefficient of determination (R^2^), adjusted R^2^, and lack-of-fit tests.

## 3. Results

### 3.1. Thermal Characterization and Solubility of Coating Materials

DSC analysis of the eleven candidate coating materials at three heating rates (5, 10, and 20 °C/min) revealed broad endothermic transitions between 100 and 165 °C (Table 1), consistent with thermally induced events associated with bound-water loss and matrix relaxation.

Peak temperatures increased systematically with heating rate in all materials, consistent with thermally activated behavior and supporting the application of the Kissinger model to estimate apparent activation energies for these transitions [19]. Because these transitions correspond to a composite endothermic event dominated by the loss of free and weakly bound water, with possible contributions from desorption of more strongly bound moisture, rearrangement of hydrogen bonding, relaxation of the amorphous phase and overlapping protein transitions, the resulting Ea values are apparent descriptors of that composite event. They are not fundamental parameters of polymer-matrix stability, are not measurements of the glass transition, and are used here only as an empirical screening criterion. It should also be noted that the DSC experiment and the spray-drying process differ substantially: a few milligrams of sample are heated in a sealed pan in which water vapour is not freely removed and the vapour pressure may rise, over heating times of minutes. A higher apparent Ea therefore cannot be assumed to translate directly into greater protection during atomization and drying. Activation energies ranged from 95.4 kJ/mol (WPC; R^2^ = 0.99) to 198.4 kJ/mol (guar gum; R^2^ = 0.882), with microcrystalline cellulose (175.3 kJ/mol; R^2^ = 0.996) and food-grade collagen (152.7 kJ/mol; R^2^ = 0.994) showing the highest resistance to thermal transition among the materials considered potentially relevant for encapsulation. Gum arabic yielded an intermediate *E*_a_ of 112.4 kJ/mol (R^2^ = 0.970); although this R^2^ was comparatively low, it was considered acceptable for the Kissinger method given the polydisperse molecular architecture of gum arabic (MW range: 250,000–2,000,000 Da), which generates partially overlapping thermal events [11]. Solubility measurements also showed marked pH-dependent behavior (Figure 1). WPC and gum arabic displayed complementary solubility profiles at pH 4.0 and 8.0, which supported their subsequent selection as a binary wall system.

### 3.2. Factorial Design for Coating Material Selection

The 3 × 2 factorial design evaluated three proteins (WPC, SPI, calcium caseinate) combined with two polysaccharides (gum arabic, OSA-modified cassava starch) as wall systems for the spray-drying microencapsulation of *L. plantarum*. Post-drying viable counts and viability values are presented in Table 2, and ANOVA results for both response variables are summarized in Supplementary Table S1.

The factorial model was statistically significant for both post-drying viable count (*p* = 0.0019; R^2^ = 0.933; adjusted R^2^ = 0.876) and viability (*p* = 0.0001; R^2^ = 0.993; adjusted R^2^ = 0.988), supporting the adequacy of the fitted models (Supplementary Table S1). Protein type (Factor A), polysaccharide type (Factor B), and their interaction (AB) significantly affected both response variables (*p* ≤ 0.023 for viable count; *p* < 0.0001 for viability), indicating that performance depended on the specific protein–polysaccharide combination. Among all combinations, WPC–gum arabic yielded the highest survival rate (97.76 ± 0.75%) and a viable count of approximately 7.1 × 10^8^ CFU/g, with the lowest inter-replicate variability, and was identified as the best-performing formulation (*p* < 0.05; Table 2, Figure 2).

SPI–OSA-modified cassava starch showed the highest mean post-drying viable count, although this treatment also exhibited greater variability than the other formulations. Calcium caseinate-based formulations, regardless of the polysaccharide used, yielded the lowest viable counts (≤2.0 × 10^8^ CFU/g) and intermediate viability values (63.9–91.8%), indicating poorer overall performance under the spray-drying conditions tested. Based on its high viability, competitive viable count, and reproducibility, WPC–gum arabic was selected as the wall system for all subsequent experiments.

### 3.3. Thermoprotectant Mixture Optimization

The D-optimal mixture design evaluated 20 experimental runs combining trehalose (A), FOS (B), glycerol (C), and inulin (D) at a total thermoprotectant concentration of 2.5% *w*/*v*. The response variable was cell survival (%) after thermal challenge at 92 ± 2 °C for 2 min, and the corresponding data for all runs are presented in Table 3. Excluding the untreated zero-time control, survival values ranged from 5.43 ± 2.02% (Run 18; trehalose 13%, FOS 63%, glycerol 13%, inulin 13%) to 14.32 ± 0.81% (Run 7; pure glycerol), corresponding to log reductions of 1.281 ± 0.166 and 0.845 ± 0.025 log CFU/mL, respectively. Pure inulin formulations (Runs 12–13) consistently yielded the lowest survival among the pure-component formulations (8.00–8.29%), while glycerol-containing formulations (Runs 1, 2, 7, 14) produced the highest values (11.7–14.3%), identifying glycerol as the single component most strongly associated with post-thermal survival. Formulations containing both trehalose and glycerol, either alone or in combination with FOS and inulin (Runs 14, 16, and 20), also showed comparatively high survival values, supporting the relevance of this region of the mixture space for further optimization.

**Figure 3:**
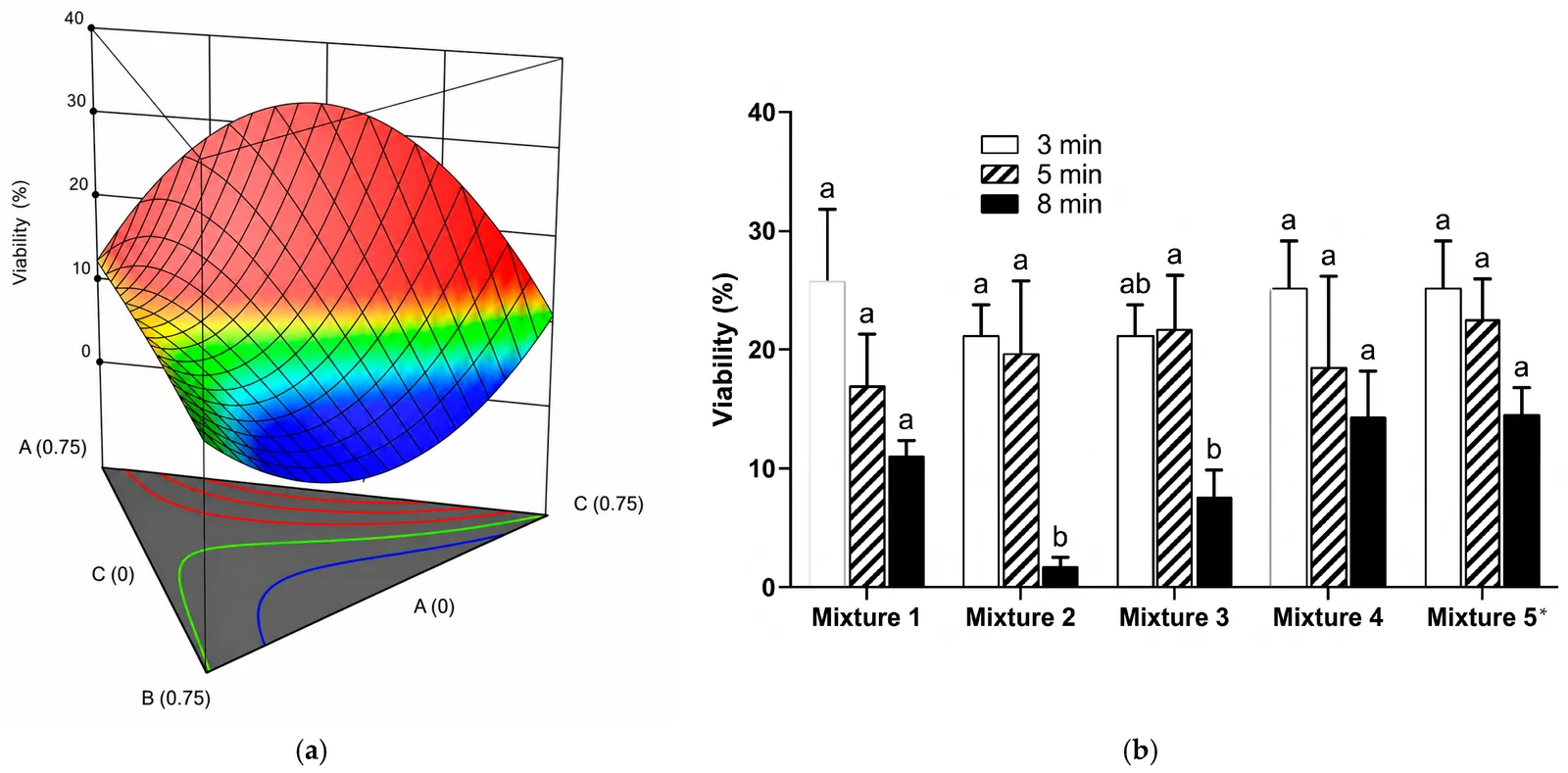
Optimization of thermoprotectant formulation for *Lactiplantibacillus plantarum* microencapsulation using a D-optimal mixture design. (**a**) Three-dimensional response surface plot of the special cubic Scheffé polynomial model showing predicted post-thermal viability (%) as a function of trehalose (A) and glycerol (C) fractions; the remaining components (FOS, B; inulin, D) were held at their centroid values. The color scale indicates predicted viability, from red (higher viability) to blue (lower viability). The response maximum is located in the region of high glycerol and intermediate trehalose proportions. (**b**) Experimental validation of the five candidate formulations (Mixtures 1–5) generated by numerical optimization, showing *L. plantarum* viability (%) after thermal challenge at 92 ± 2 °C for 3 min (□), 5 min (▨), and 8 min (■). Error bars represent standard deviation (*n* = 3). Different lowercase letters above the bars indicate significant differences among formulations within the same exposure time (*p* < 0.05; Tukey’s HSD test). Asterisk (*) indicates the selected optimal formulation (Mixture 5) based on maximum viability across all exposure times. Formulation composition per 2.5% (*w*/*v*) total thermoprotectant: Mixture 1—trehalose 60.0%, FOS 10.0%, glycerol 5.0%, inulin 25.0%; Mixture 2—trehalose 70.0%, FOS 2.5%, glycerol 2.5%, inulin 25.0%; Mixture 3—trehalose 50.0%, FOS 20.0%, glycerol 5.0%, inulin 25.0%; Mixture 4—trehalose 50.0%, FOS 25.0%, glycerol 0.0%, inulin 25.0%; Mixture 5*—trehalose 29.7%, FOS 19.8%, glycerol 25.7%, inulin 24.8%.

The special cubic Scheffé polynomial model was fitted to the experimental data and validated using ANOVA (Supplementary Table S2). The overall model was statistically significant (*p* = 0.009), and the non-significant Lack-of-fit (*p* = 0.162) supported the adequate model fit without systematic deviation across the design space. The linear mixture terms were jointly significant (*p* = 0.007), indicating differential individual contributions of the four components to the thermoprotective response. At the binary interaction level, FOS–glycerol (BC; *p* = 0.031), FOS–inulin (BD; *p* = 0.019), and trehalose–inulin (AD; *p* = 0.041) were statistically significant, while trehalose–FOS (AB; *p* = 0.227), trehalose–glycerol (AC; *p* = 0.270), and glycerol–inulin (CD; *p* = 0.273) were not. These three terms were nevertheless retained because the special cubic Scheffé model is hierarchical: the significant three-component terms ABC, ACD, and BCD require the presence of the lower-order terms nested within them, and their removal would yield a non-hierarchical model whose coefficients are no longer invariant to the choice of component reference in the simplex coordinate system. The pattern of significant and non-significant pairs therefore indicates selective synergistic or antagonistic pairing effects within a model whose term structure is fixed by hierarchy rather than by stepwise selection. The ternary interactions ABC (*p* = 0.017), ACD (*p* = 0.002), and BCD (*p* = 0.022) were all significant, indicating that higher-order interactions contributed to the response. The model explained 91.9% of the observed variance (R^2^ = 0.919; adjusted R^2^ = 0.781), and the difference between R^2^ and adjusted R^2^ (0.138) is consistent with the inclusion of higher-order polynomial terms necessary to capture the nonlinear behavior of the quaternary mixture space; importantly, the non-significant lack-of-fit confirms that this gap does not reflect systematic model inadequacy. The predicted R^2^ was 0.234, and the difference relative to the adjusted R^2^ indicates that the model, while adequate for describing the mixture space and locating the optimal region, has limited extrapolative capability. This behavior is expected for a ten-term special cubic model fitted to twenty runs: the resulting seven residual degrees of freedom give each design point high leverage, which inflates the leave-one-out PRESS statistic on which the predicted R^2^ is based. For this reason, the optimum was confirmed experimentally rather than relying on model prediction alone (Figure 3b). Residual diagnostics are provided in Supplementary Figure S1; no observation exceeded the Bonferroni outlier limits (±5.60) and the residual variance was homogeneous across the predicted range.

Numerical optimization using a desirability function identified five candidate formulations (Mixtures 1–5), which were then experimentally validated at three thermal exposure times (3, 5, and 8 min at 92 °C). Predicted and observed responses are depicted in Figure 3. The three-dimensional response surface (Figure 3a) illustrates the relationship between trehalose and glycerol fractions and predicted post-thermal survival, with the response maximum located in the region of high glycerol and intermediate trehalose proportions. Experimental validation of the five candidate formulations (Figure 3b) confirmed that Mixture 5 (trehalose 29.7%, FOS 19.8%, glycerol 25.7%, inulin 24.8%) achieved the highest and most consistent post-thermal survival across all three exposure times, with no statistically significant decline between 3 and 8 min (*p* > 0.05), indicating that the protective effect is not lost as the thermal load increases. It should be noted that the absolute survival values in this design remained low (5–14%), so the relevant outcome is the stability of the protective effect across exposure times rather than a high intrinsic heat resistance. Mixture 5 was therefore selected as the optimal thermoprotectant formulation for subsequent microcapsule characterization.

### 3.4. Characterization of Optimized Microcapsule

**Figure 4:**
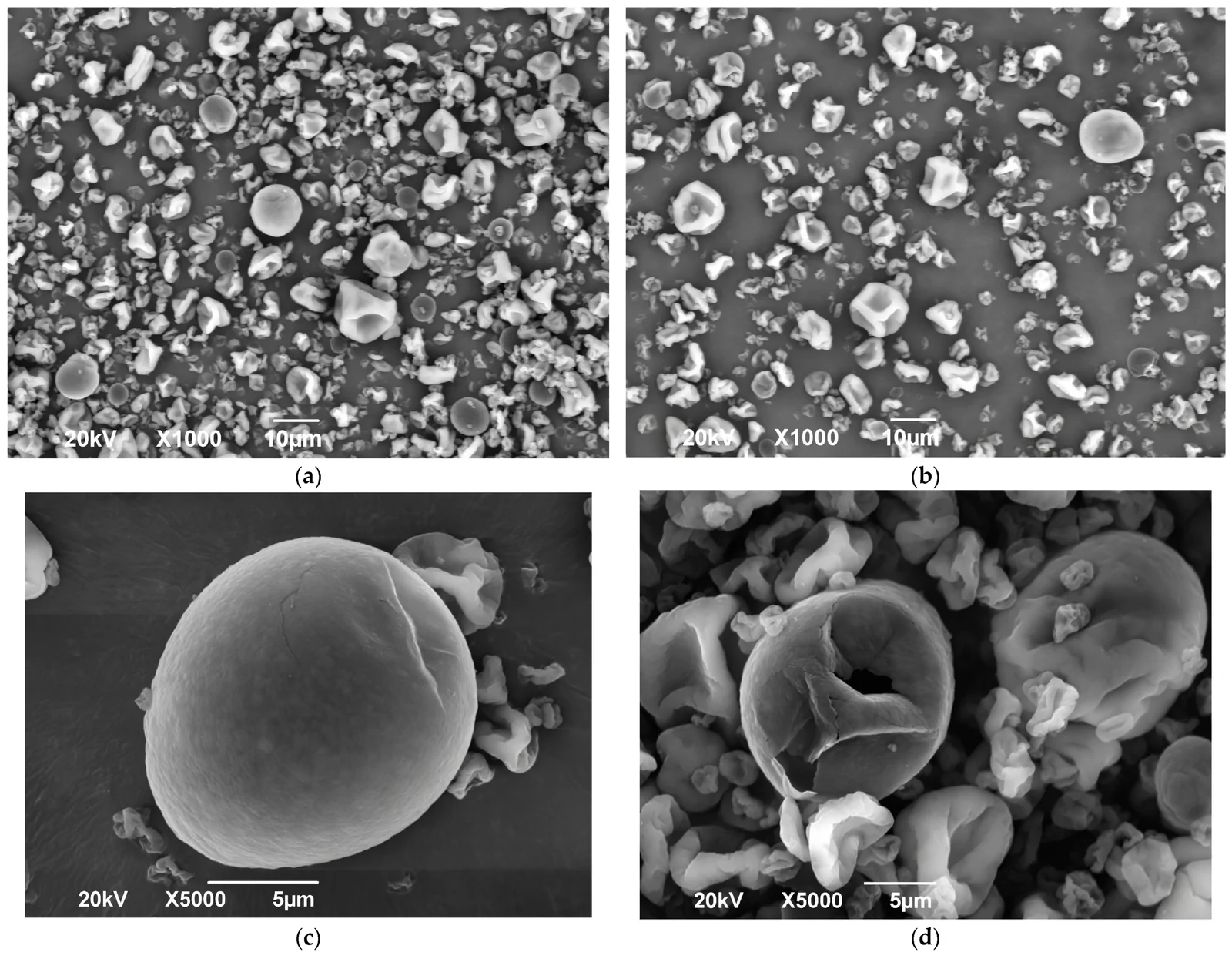
Scanning electron micrographs of optimized *L. plantarum* microcapsules (WPC–gum arabic/Mixture 5). (**a**,**b**) ×1000 magnification (scale bar = 10 µm); (**c**,**d**) ×5000 magnification (scale bar = 5 µm). Panels (**c**,**d**) show, respectively, a spherical capsule with smooth intact surface and capsules exhibiting characteristic drying-induced surface indentations.

The physicochemical and microbiological characterization of WPC–gum arabic microcapsules with and without the optimized thermoprotectant formulation (Mixture 5) is presented in Table 4 and Figure 4. Thermoprotectant-loaded microcapsules yielded a post-drying viable count of 2.4 × 10^11^ CFU/g versus 8.5 × 10^8^ CFU/g for unprotected capsules (Δ = +2.45 log CFU/g; *p* < 0.05), with survival rates of 73.16 ± 0.64% and 58.42 ± 2.15%, respectively. Following thermal challenge at 92 °C for 2 min, viable counts declined by 1.54 log cycles for thermoprotectant-loaded capsules (from 2.4 × 10^11^ to 6.9 × 10^9^ CFU/g; thermal resistance: 2.86%) versus 2.60 log cycles for unprotected capsules (from 8.5 × 10^8^ to 2.1 × 10^6^ CFU/g; thermal resistance: 0.25%), with the post-thermal count for thermoprotectant-loaded capsules remaining of the order of 10^9^ CFU/g of powder. No comparison with the FAO/WHO recommended intake is made here, because that recommendation is expressed per administered dose whereas the experimental endpoint of this work is expressed per gram of powder; establishing the delivered dose would require defining the intended serving mass of the final product, which falls outside the scope of this study. It should be emphasized that the absolute thermal resistance of both formulations remains low: the thermoprotectant blend reduced the loss from 2.60 to 1.54 log cycles, an eleven-fold relative improvement in the retained fraction, but approximately 97% of the initial population was still inactivated. The technological relevance of the optimized formulation therefore derives from the combination of a high post-drying load and a reduced log loss, which together leave a useful residual count after processing, rather than from a high intrinsic heat resistance. Water activity was below the critical stability limit (aw < 0.25) for both formulations (0.23 ± 0.02 and 0.19 ± 0.02, respectively).

Moisture content was higher in Mixture 5 capsules than in unprotected capsules (9.49 ± 0.25% vs. 8.12 ± 0.81%), consistent with the presence of hygroscopic carbohydrates such as FOS and inulin. Mean particle size did not differ significantly between thermoprotectant-loaded and unprotected microcapsules (D_4,3_ = 10.43 ± 2.02 and 12.18 ± 1.84 µm, respectively; *p* > 0.05). In contrast, thermoprotectant-loaded microcapsules showed improved flow properties, with a lower Carr Index (18.5 ± 2.1% vs. 24.3 ± 2.8%) and Hausner Ratio (1.20 ± 0.03 vs. 1.32 ± 0.05). SEM micrographs suggested that unprotected microcapsules presented irregular, collapsed morphologies at ×1000 magnification, while thermoprotectant-loaded microcapsules were predominantly spherical with smooth intact surfaces and only superficial concavities consistent with spray drying-induced shrinkage, with no evidence of cracks or wall discontinuities [28].

### 3.5. In Vitro Gastrointestinal Survival

**Figure 5:**
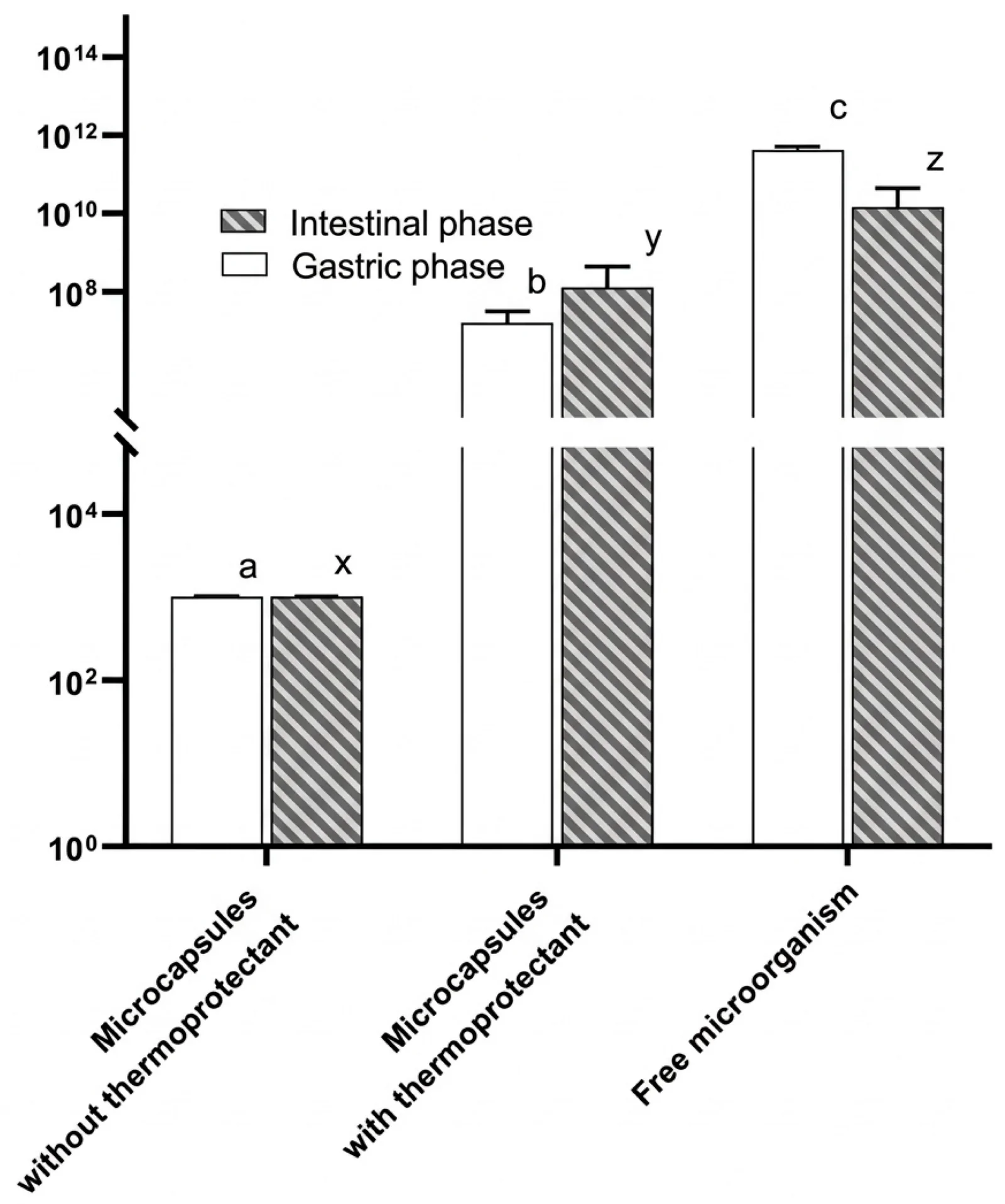
In vitro gastrointestinal survival of *L. plantarum* under simulated INFOGEST 2.0 conditions. Viable counts (CFU/mL of digesta) are shown for the gastric phase (pH 3.0, pepsin, 120 min; open bars) and intestinal phase (pH 7.0, pancreatin + bile salts, 120 min; hatched bars) for three treatments: unprotected microcapsules (WPC–gum arabic without thermoprotectant), thermoprotectant-loaded microcapsules (WPC–gum arabic + Mixture 5), and free *L. plantarum* cells. Values represent mean ± SD of three independent digestion runs (*n* = 3). Superscript letters denote statistically significant differences among treatments within each phase (one-way ANOVA and Tukey’s HSD test on log_10_-transformed counts, *p* < 0.05): a–c for the gastric phase and x–z for the intestinal phase; treatments sharing a letter do not differ significantly. Gastric and intestinal counts within each treatment were compared by a paired Student’s *t*-test (*p* < 0.05).

Thermoprotectant incorporation governed survival throughout simulated digestion (Figure 5). Microcapsules produced without thermoprotectant were effectively inactivated, retaining only 1.0 × 10^3^ ± 1.0 × 10^1^ CFU/mL of digesta after the gastric phase and the same order of magnitude after the intestinal phase (1.0 × 10^3^ ± 1.0 × 10^1^ CFU/mL of digesta). Loading the wall system with Mixture 5 raised survival by four orders of magnitude: thermoprotectant-loaded microcapsules delivered 1.5 × 10^7^ ± 1.6 × 10^7^ CFU/mL of digesta after the gastric phase and 1.2 × 10^8^ ± 3.0 × 10^8^ CFU/mL of digesta after the intestinal phase, a further one-order-of-magnitude gain across intestinal transit. Free *L. plantarum* cells held the highest absolute counts, 4.0 × 10^11^ ± 1.0 × 10^11^ CFU/mL of digesta in the gastric phase and 1.4 × 10^10^ ± 3.0 × 10^10^ CFU/mL of digesta in the intestinal phase, and were the only treatment that lost viability during transit, a decrease of 1.5 log cycles between phases. For some treatments, the standard deviation expressed in linear units is of the same order as, or larger than, the mean. This is a consequence of describing log-normally distributed plate-count data on an arithmetic scale: when the viable counts of independent replicates span an order of magnitude, the arithmetic standard deviation can exceed the arithmetic mean without any individual observation being aberrant. The dispersion is biological rather than analytical and reflects differences between independent digestion runs in matrix disintegration, capsule rehydration, and enzyme accessibility; on the logarithmic axis of Figure 5 it corresponds to a symmetric interval of a few tenths of a log unit. The differences between treatments span four to eight orders of magnitude and are therefore substantially larger than the within-treatment dispersion, so the comparisons reported here do not depend on the precise magnitude of the standard deviations.

Across the full transit, viable counts ranked free cells > thermoprotectant-loaded microcapsules > unprotected microcapsules in both phases (Figure 5). Only the encapsulated formulations increased their counts from the gastric to the intestinal phase, whereas free cells declined by 1.5 log cycles. The difference between thermoprotectant-loaded and unprotected microcapsules widened from 4.2 log cycles in the gastric phase to 5.1 log cycles in the intestinal phase.

## 4. Discussion

### 4.1. Thermal Characterization and Solubility of Coating Materials: Thermophysical Basis for Rational Selection

The thermophysical characterization of eleven candidate coating materials by DSC and Kissinger analysis provided the basis for the material-selection strategy used in this study, extending the criterion originally proposed by Barreto et al. [16], who demonstrated that higher *E*_a_ values in carbohydrate polymer blends directly correlated with greater core protection. The broad endothermic transitions observed between 100 and 165 °C are consistent with composite thermal events involving bound-water loss and macromolecular relaxation, as expected for amorphous biopolymer systems in which residual moisture acts as a plasticizer [10,11]. It should be noted that the *E*_a_ values derived from these transitions represent the energy barrier for the coupled desorption–relaxation process, not for covalent bond degradation of the polymer backbone. Nevertheless, this parameter may still be functionally informative, since materials with higher apparent *E*_a_ may be hypothesized to resist structural relaxation for longer periods at elevated temperatures, potentially providing thermal shielding during the transient high-temperature exposure inherent to spray drying [19]; this remains a hypothesis, since the glass transition temperature was not measured (Section 3.1). The systematic shift of peak temperatures with increasing heating rate supported the application of the Kissinger model across all candidates [19]. For gum arabic, the fitted R^2^ remained acceptable for comparative purposes and may reflect the well-known polydispersity of this material, which can broaden and partially overlap thermal events [11]. Despite their high *E*_a_ values (198.4 kJ/mol for guar gum and 175.3 kJ/mol for microcrystalline cellulose), neither material was suitable for spray drying at 10% (*w*/*v*) under the conditions used in this study because both formed highly viscous or poorly dispersible systems that were incompatible with stable and uniform atomization [34]. This confirms that thermal transition energy is a necessary but insufficient selection criterion when evaluated in isolation, given that aqueous solubility, food-grade regulatory status, emulsifying capacity, and film-forming kinetics must be assessed concurrently [7]. Among protein candidates satisfying all functional criteria, WPC (*E*_a_ = 95.4 kJ/mol), SPI (*E*_a_ = 118.5 kJ/mol), and calcium caseinate (*E*_a_ = 127.8 kJ/mol) were selected because they represent systems with distinct thermal and interfacial properties: WPC combines whey protein-driven surface film formation with a lactose-rich amorphous phase that contributes to the overall *T*_g_ of the dried matrix; SPI provides reduced solubility under acidic conditions (pI ≈ 4.5), potentially limiting premature cell release in acidic food matrices; and calcium caseinate offers a disordered micellar structure with higher intrinsic thermal transition energy but slower film-forming kinetics [15,16]. WPC is a multicomponent system containing β-lactoglobulin, α-lactalbumin, bovine serum albumin, immunoglobulins, and residual lactose (typically 35–55% depending on the concentrate grade), and its thermal behavior in DSC reflects the superposition of whey protein denaturation (onset ~65 °C for β-lactoglobulin) and lactose glass transition events. The relatively low apparent *E*_a_ of WPC should therefore not be interpreted as intrinsically poor thermal suitability. Rather, its behavior likely reflects overlapping protein and lactose-related transitions, and the amorphous lactose fraction may have contributed additional stabilization to the dried matrix [35,36]. Among the polysaccharides, gum arabic was particularly attractive because of its surface-active proteinaceous fraction (approximately 2% by weight) and its functional suitability for food applications. OSA-modified cassava starch (*E*_a_ = 98.6 kJ/mol; contact angle 76.10°) was also considered a promising candidate because of its amphiphilic character and potential interfacial functionality. Solubility measurements at pH 4.0 and 8.0 (Figure 1) confirmed differential pH-dependent profiles for the five candidates selected for factorial evaluation. The selection of pH 4.0, proximate to the isoelectric point of the major whey protein fractions (β-lactoglobulin pI ≈ 5.2; α-lactalbumin pI ≈ 4.2), and pH 8.0, representing mildly alkaline conditions encountered in the distal small intestine, was designed to capture the extremes of protein solubility behavior relevant to food processing and gastrointestinal transit. At pH 4.0, WPC exhibited reduced solubility consistent with charge neutralization and protein aggregation near the pI, which would contribute to wall integrity in acidic food matrices (pH 3.5–4.5) and during the initial stages of gastric transit. At pH 8.0, increased solubility was observed for both WPC and gum arabic, consistent with electrostatic repulsion above the pI, which would favor controlled wall dissolution and probiotic release under intestinal alkaline conditions [32]. These combined thermophysical and solubility criteria provided the quantitative rationale for advancing WPC, SPI, and calcium caseinate as protein factors and gum arabic and OSA-modified cassava starch as polysaccharide factors to the factorial design.

### 4.2. Protein–Polysaccharide Combination Screening and Thermoprotectant Optimization

#### 4.2.1. Wall System Selection: Protein–Polysaccharide Synergy Under Spray Drying Conditions

The significant AB interaction identified in the factorial ANOVA (*p* = 0.002 for viable count; *p* < 0.0001 for viability; Supplementary Table S1) indicates that protective performance was not simply additive but depended on the specific protein–polysaccharide combination formed during atomization and drying. This pattern is consistent with the multilayer wall-formation model described by Gharsallaoui et al. [7], in which proteins dominate early interfacial film formation, whereas polysaccharides contribute to water retention, matrix cohesion, and the thermal stability of the consolidated wall.

The superior performance of WPC–gum arabic (post-drying survival = 97.76 ± 0.75%; viable count = 7.1 × 10^8^ CFU/g; Table 2) is consistent with at least two complementary mechanisms associated with this protein–polysaccharide pair. First, the whey protein fraction of WPC likely promotes rapid interfacial film formation during the early stages of drying, generating a continuous viscoelastic film at the droplet boundary that limits heat transfer to the particle interior before lethal temperatures are reached. This interpretation is supported by previous studies in whey-protein systems showing reduced membrane damage relative to SPI- or caseinate-based matrices under comparable drying conditions [36]; while WPC contains additional non-protein components (lactose, fat), the same protein-driven film formation mechanism is expected to operate, with the additional contribution of amorphous lactose to the glass transition temperature of the resulting matrix [35]. Second, gum arabic likely contributes a polysaccharide-rich matrix with favorable film-forming and water-binding properties, helping to stabilize the wall structure during drying. In addition, the residual lactose present in WPC (~35–55%, depending on concentrate grade) may have provided a further stabilization pathway through its contribution to the amorphous phase of the dried matrix. Amorphous lactose, with a *T*_g_ of approximately 101 °C, can act as a co-vitrifier that increases the effective *T*_g_ of the wall matrix, potentially extending the protective glassy state to higher temperatures and longer exposure times. This interpretation is consistent with the high survival observed, although the precise contribution of each WPC component to the overall thermal protection cannot be deconvoluted from the present experimental design and warrants further investigation using compositionally defined protein isolates. The poorer performance of calcium caseinate formulations (Table 2), despite their comparatively high Kissinger activation energy (127.8 kJ/mol; Table 1), indicates that thermal transition parameters alone do not adequately capture encapsulation performance. This observation reinforces the need for a multicriteria formulation strategy in which thermal behavior is interpreted together with capsule morphology, barrier properties, and storage conditions, as highlighted by recent reviews on the microencapsulation of food bioactives [37]. Caseinate consists of open, disordered micellar aggregates whose rheological behavior under atomization conditions is predominantly viscous rather than viscoelastic [16], resulting in slower and less uniform film formation at the droplet surface, which allows thermal penetration to the particle interior before a continuous protective barrier is established. SPI–OSA starch produced the highest mean viable count (9.8 × 10^9^ CFU/g), but it also showed the greatest inter-replicate variability (CV = 3.02%; Table 2). This reduced reproducibility may be related to structural and interfacial heterogeneity previously described for OSA-modified starch [38]. From a formulation perspective, this higher variability weakens the practical attractiveness of SPI–OSA starch despite its high mean count, particularly in comparison with the more reproducible WPC–gum arabic system. WPC–gum arabic was therefore selected as the optimal wall system. Because the two responses of the factorial design ranked different treatments first, the selection was made against an explicit multi-response criterion rather than on viable count alone. SPI–OSA-modified cassava starch gave the highest absolute post-drying count, but it was not selected for three reasons. First, its post-drying survival was substantially lower (66.11% versus 97.76%), indicating that its high absolute count reflects the load of the feed rather than protection during drying. Second, its between-replicate variability was the largest of the six combinations, which compromises reproducibility at scale. Third, the pH-dependent solubility profile of OSA-modified cassava starch was less favourable across the acidic and alkaline conditions tested, which is directly relevant to the intended gastrointestinal application. Survival, reproducibility, and pH-dependent solubility were therefore weighted above absolute viable count, since a formulation that preserves a high fraction of the cells it receives is more transferable than one whose apparent performance depends on the concentration of the inoculum.

#### 4.2.2. Thermoprotectant Optimization

The significant ternary interactions identified in the D-optimal mixture design (ABC *p* = 0.017; ACD *p* = 0.002; BCD *p* = 0.022; Supplementary Table S2) confirm that thermoprotective performance at 92 °C is governed by multicomponent synergies operating through mechanistically distinct but spatially complementary cellular protection pathways. This interpretation is consistent with the dual-mechanism framework proposed by Crowe and colleagues, which combines water replacement at membrane interfaces with vitrification-based immobilization of the intracellular milieu [39], and with its later extension to multicomponent thermoprotectant systems. Trehalose (29.7% in Mixture 5) likely contributed through intracellular vitrification. As a non-reducing disaccharide with an anhydrous *T*_g_ of 115.9 °C, it can form cooperative hydrogen-bonding networks that immobilize lipid bilayers and cytoplasmic proteins in a glassy matrix, thereby reducing thermally induced conformational changes during rapid dehydration [13,17]. Trehalose has also been proposed to replace structural water molecules at the polar headgroup region of phospholipids, helping to preserve bilayer integrity during the dehydration associated with both spray drying and thermal challenge [39]. Glycerol (25.7%) may have contributed through a faster, membrane-directed effect. As a low-molecular-weight polyol (MW 92.09 g/mol), it can interact with phospholipid head groups during the initial stages of dehydration, potentially limiting lateral phase separation before trehalose-mediated vitrification becomes dominant [39]. This temporal complementarity is consistent with the higher individual survival values observed for glycerol monoformulations (Runs 2 and 7; 11.7–14.3%; Table 3) relative to trehalose monoformulations (Runs 3 and 8; 10.6–11.4%; Table 3), as well as with the improved performance observed when both were combined. However, glycerol is also expected to depress the *T*_g_ of the amorphous matrix because of its low molecular weight and plasticizing effect. The fact that glycerol-containing formulations still showed higher survival under the acute thermal challenge used here (92 °C, 3–8 min) suggests that membrane-associated stabilization may have outweighed any *T*_g_-depressing effect under these conditions. Whether this balance would shift during prolonged storage at ambient temperature, where matrix mobility becomes more critical, remains to be determined. FOS (19.8%) and inulin (24.8%) likely contributed primarily at the extracellular level through osmotic buffering and matrix-forming effects. Their high degree of polymerization (FOS: DP 3–10; inulin: up to 60) would also be expected to limit membrane penetration, which is consistent with the comparatively low survival of pure inulin formulations (Runs 12–13; 8.0–8.3%; Table 3) [26]. Within the quaternary mixture, FOS and inulin likely acted as extracellular matrix-forming components that slowed moisture migration during thermal challenge and contributed to the overall *T*_g_ of the dried system through their relatively high molecular weight, thereby complementing the protection associated with trehalose and glycerol. The non-significant lack-of-fit (*p* = 0.162; Supplementary Table S2) together with the relatively high model fit (R^2^ = 0.919) indicates that the special cubic Scheffé polynomial adequately describes the experimental data within the design space. The low predicted R^2^ (0.234), however, indicates limited extrapolative capability, so the model should be regarded as a descriptive and screening tool rather than a predictive one. Accordingly, the selection of Mixture 5 as the most promising thermoprotectant formulation rests primarily on its experimental validation under the acute thermal challenge conditions tested.

These mechanistic attributions are inferential rather than demonstrated, because the glass transition temperature of the complete optimized system was not measured in this work. The 2.5% (*w*/*v*) thermoprotectant concentration refers to the feed; relative to the 12.5% (*w*/*v*) total solids of the thermoprotectant-loaded feed, the blend accounts for approximately 20% (*w*/*w*) of the dry matter of the resulting powder. This is a compositionally relevant fraction, but it does not by itself establish that a glassy state was attained under the drying conditions used. In the absence of direct thermal or spectroscopic evidence, vitrification should therefore be regarded as one of several plausible contributions, alongside water replacement at membrane interfaces, local osmotic stabilization of the extracellular environment, and direct membrane-associated protection by the low-molecular-weight components. Discriminating among these mechanisms would require modulated DSC or dynamic mechanical analysis of the complete system, together with membrane-integrity assays.

A further limitation concerns the structure of the optimization itself. The thermoprotectant blend was optimized in suspension, independently of the spray-drying step and of the wall-material composition, so the identified optimum is the optimum for resistance to the thermal challenge applied to the suspension rather than necessarily the optimum for the final microcapsule. Thermoprotectant composition can modify feed rheology and surface activity, and thereby atomization, drying kinetics, particle formation, moisture distribution, and the final matrix structure. The experimental validation of Mixture 5 in the complete spray-dried system (Table 4, Figure 4) mitigates this limitation but does not remove it; a fully coupled optimization, in which wall composition, thermoprotectant composition, and process variables are varied together, remains an open task.

### 4.3. Characterization of the Optimized Microcapsule and Gastrointestinal Resistance

#### 4.3.1. Physical Structure and Powder Functionality

The 2.45 log CFU/g improvement in viable count obtained with the thermoprotectant formulation (2.4 × 10^11^ vs. 8.5 × 10^8^ CFU/g; Table 4) is consistent with the combined contribution of multiple protective mechanisms operating during spray drying. During the early stages of drying, glycerol may contribute through rapid membrane-associated stabilization, whereas trehalose may progressively replace hydration water and favor formation of a glassy matrix as dehydration proceeds [39]. In parallel, the amorphous lactose fraction of WPC may also contribute to the overall *T*_g_ of the matrix, providing an additional vitrification pathway that complements trehalose-associated glass formation. This multicomponent interpretation is consistent with the observation that no single component matched the survival achieved with the quaternary mixture.

SEM micrographs of the optimized microcapsules (Figure 4) show predominantly spherical particles together with surface concavities characteristic of spray-dried materials, consistent with shrinkage during drying rather than extensive wall disruption. These images describe particle morphology and wall continuity only; they do not provide evidence of the spatial distribution or embedding of the bacterial cells within the matrix.

Mean particle size was not affected by thermoprotectant incorporation (D_4,3_ = 10.43 ± 2.02 µm versus 12.18 ± 1.84 µm for unprotected microcapsules; *p* > 0.05), indicating that the addition of 2.5% (*w*/*v*) low-molecular-weight solutes modified the composition of the drying droplet without altering its atomization behavior. This outcome is expected because, at a constant wall-solids content of 10% (*w*/*v*) and a difference of only 2.5 percentage points in total feed solids, particle size in two-fluid nozzle spray drying is governed mainly by atomization air-to-feed ratio, feed flow rate, and feed viscosity rather than by the identity of the dissolved solutes [7]. Both formulations lie within the 10–20 µm range commonly reported for laboratory-scale spray-dried probiotic powders, a size that is large enough to accommodate *L. plantarum* cells (approximately 1 × 3 µm) within a continuous wall matrix while remaining below the threshold at which particles are perceived as grittiness in fluid foods, which is technologically relevant for the intended incorporation into beverages and dairy matrices. The absence of a size difference also constrains the mechanistic interpretation of the viability data: because surface-area-to-volume ratio and wall diffusional path length were comparable between formulations, the superior thermal and gastrointestinal performance of the thermoprotectant-loaded capsules is more consistently explained by compositional and vitrification effects than by particle geometry. The relatively wide standard deviations reflect the broad size distributions inherent to two-fluid nozzle atomization, and narrowing this distribution through nozzle and feed-viscosity optimization remains an opportunity for improving dosing accuracy at larger scale.

Water activity values below 0.25 for both microcapsule formulations (Table 4) are consistent with limited molecular mobility in the powder and with improved physicochemical stability during storage [10]. The modest increase in moisture content relative to the control (9.49 vs. 8.12%) is consistent with the hygroscopic nature of FOS and inulin components in Mixture 5 and is unlikely to compromise microbiological stability at the measured aw values. The lower Carr Index (18.5 ± 2.1 vs. 24.3 ± 2.8%) indicates improved flowability, shifting the material from the “fair” to the “good” flowability range according to conventional Carr Index classification [40]. This improvement in flow behavior is technologically relevant because it would be expected to facilitate handling and more consistent volumetric dosing in continuous food processing operations [31].

#### 4.3.2. Gastrointestinal Resistance

The in vitro digestion results (Figure 5) showed that thermoprotectant loading, rather than encapsulation alone, governed viable-cell recovery across the three experimental conditions (free cells, unprotected microcapsules, and thermoprotectant-loaded microcapsules), indicating that the thermoprotectant blend, and not the wall matrix by itself, accounted for the recovery observed. Unprotected microcapsules showed the greatest loss during the gastric phase (pH 3.0, pepsin 2000 U/mL, 120 min), with recovery approximately 4 logarithmic cycles below that of the thermoprotectant-loaded capsules. This behavior is consistent with the combined effects of acid stress and proteolytic challenge under simulated fasting conditions [32].

Unprotected WPC–gum arabic capsules did not provide gastric protection relative to free cells; their recovery was several logarithmic cycles lower. This indicates that reduced solubility of whey protein fractions under acidic conditions was not, by itself, sufficient to shield the encapsulated cells. Although charge neutralization below the isoelectric point of β-lactoglobulin (approximately 5.2) would be expected to promote protein aggregation and reduce wall permeability, this mechanism was evidently insufficient to shield the encapsulated cells from acid and enzymatic exposure. Progressive pepsin-mediated hydrolysis during the 120 min gastric incubation would be expected to increase wall permeability over time, while the absence of intracellular thermoprotectants leaves cells more vulnerable to damage once the barrier is partially compromised. The increase in counts recorded for both encapsulated formulations between the gastric and intestinal phases reflects wall dissolution at pH 7.0 in the presence of pancreatin and bile salts, which may release cells retained within the matrix, together with the recovery of culturability in sublethally acid-injured cells after neutralization. The intestinal counts therefore quantify the fraction of the population effectively delivered to the site of absorption, and they establish the thermoprotectant-loaded system as the formulation that couples matrix protection with release at the intended target.

Thermoprotectant-loaded microcapsules maintained high viable counts after the gastric phase, with no statistically significant difference between gastric and intestinal measurements (paired *t*-test, *p* > 0.05; Figure 5). This stronger performance relative to unprotected capsules is consistent with a multibarrier interpretation in which the WPC–gum arabic wall acts as the first barrier against acid and pepsin exposure, while trehalose and glycerol provide an additional intracellular or membrane-associated layer of protection. Even if partial proton penetration occurs during extended gastric incubation, these thermoprotectants may help preserve membrane and protein stability under conditions that would otherwise be highly damaging [13,39]. It should be noted that free cells retained the highest absolute counts in both digestive phases (Figure 5), so the present data do not demonstrate that encapsulation increased absolute viable-cell recovery relative to free cells. The comparison supported by this experimental design is the one between unprotected and thermoprotectant-loaded microcapsules, which differed by 4.2 and 5.1 logarithmic cycles in the gastric and intestinal phases, respectively.

Intestinal counts for thermoprotectant-loaded capsules (Figure 5) remained of the order of 10^8^ CFU/mL of digesta. This result is consistent with sufficient wall dissociation under the intestinal conditions tested (pH 7.0, pancreatin, and bile salts) to allow delivery of a high viable cell population to the intestinal phase. The increase in viable counts from the gastric to the intestinal phase for both encapsulated formulations warrants careful interpretation. Two non-mutually exclusive mechanisms may account for this pattern: (i) pH-triggered dissolution of the wall at pH 7.0 may release previously entrapped but viable cells that were not fully recovered during the gastric phase, and (ii) neutralization of the acidic environment may allow sublethally injured cells to recover culturability. The present experimental design does not allow discrimination between these mechanisms, and complementary approaches such as viability staining or flow cytometry would be needed to distinguish between viable, injured, and dead cell populations. Even with this limitation, the overall pattern remains consistent with the central design logic of this study: pH-dependent wall solubility can be exploited to combine greater resistance under gastric conditions with increased release under intestinal-like conditions. Although the screening pH values (4.0 and 8.0) do not exactly reproduce the gastric (pH 3.0) and intestinal (pH 7.0) conditions of the INFOGEST protocol, they captured the same directional behavior of lower solubility under acidic conditions and higher solubility under neutral-to-alkaline conditions.

## 5. Conclusions

A property-driven approach integrating Kissinger thermal transition analysis as an empirical screening descriptor, pH-dependent solubility profiling, and sequential experimental design guided the development of spray-dried *L. plantarum* microcapsules with improved thermal and gastrointestinal stability. Thermal transition energy (*E*_a_) alone was not a sufficient selection criterion, but interpreted together with film-forming behavior and pH-dependent solubility it identified WPC–gum arabic as the most suitable binary wall system (97.76 ± 0.75% survival; significant protein–polysaccharide interaction, *p* < 0.0001). Thermoprotection at 92 °C depended on higher-order multicomponent interactions (*p* ≤ 0.022), and the optimized quaternary blend (trehalose 0.297, glycerol 0.257, FOS 0.198, inulin 0.248; total 2.5% *w*/*v*) improved post-drying viability by 2.45 log CFU/g and maintained viable counts of the order of 10^7^ to 10^8^ CFU/mL of digesta throughout simulated INFOGEST 2.0 digestion. The comparison with the unprotected control is equally informative: WPC–gum arabic microcapsules without internal protectants were effectively inactivated during the gastric phase, retaining counts of the order of 10^3^ CFU/mL of digesta. The protein–polysaccharide wall therefore acts mainly as a physical barrier and as a vehicle for intestinal release, and is not by itself sufficient for gastric survival; the internal osmotic and membrane-stabilizing protectants are what make gastric transit survivable. It should also be noted that free cells of this acid-tolerant strain retained higher absolute counts than the encapsulated systems, so the gastrointestinal benefit demonstrated here is relative to unprotected microcapsules rather than to free cells.

Direct measurement of the Tg of the complete optimized system, clarification of the contribution of the WPC lactose fraction, and long-term storage and food-matrix validation remain necessary. Even so, the framework offers a transferable, experimentally validated alternative to empirical material selection for the design of thermally stable probiotic ingredients.

## Figures and Tables

**Table 1:** Differential scanning calorimetry (DSC) parameters of coating material candidates at three heating rates (5, 10, and 20 °C/min).

Polymer	Rate (°C/min)	ΔH (J/g)	Onset (°C)	Endset (°C)	Peak Width (°C)	*E*_a_ (kJ/mol)	R^2^ (Kissinger)
Gum arabic	5	−280.55 ± 5.61 ^a^	48.01 ± 0.96 ^ef^	142.26 ± 2.85 ^ab^	55.31 ± 1.11 ^a^	112.4	0.9702
	10	−277.17 ± 9.42 ^a^	52.37 ± 1.78 ^e^	151.73 ± 5.16 ^ab^	58.10 ± 1.98 ^ab^		
	20	−204.02 ± 9.59 ^b^	67.57 ± 3.18 ^bc^	162.38 ± 7.63 ^a^	63.51 ± 2.98 ^a^		
Sodium caseinate	5	−101.56 ± 2.64 ^de^	81.41 ± 2.12 ^b^	147.27 ± 3.83 ^a^	51.81 ± 1.35 ^ab^	145.2	0.931
	10	−101.20 ± 5.16 ^e^	72.35 ± 3.69 ^b^	148.50 ± 7.57 ^ab^	52.80 ± 2.69 ^bcd^		
	20	−130.41 ± 3.91 ^f^	51.94 ± 1.56 ^d^	150.53 ± 4.52 ^abc^	54.65 ± 1.64 ^bc^		
Guar gum	5	−132.24 ± 5.55 ^c^	52.30 ± 2.20 ^cdef^	138.50 ± 5.82 ^abc^	52.21 ± 2.19 ^ab^	198.4	0.882
	10	−182.48 ± 10.22 ^c^	58.50 ± 3.28 ^cde^	154.23 ± 8.64 ^a^	53.43 ± 2.99 ^bcd^		
	20	−160.28 ± 3.69 ^de^	62.71 ± 1.44 ^c^	147.54 ± 3.39 ^abc^	55.86 ± 1.28 ^bc^		
Calcium caseinate	5	−75.66 ± 2.88 ^f^	55.87 ± 2.12 ^cd^	121.19 ± 4.61 ^de^	43.85 ± 1.67 ^c^	127.8	0.994
	10	−127.31 ± 5.60 ^d^	65.48 ± 2.88 ^bc^	126.23 ± 5.55 ^cd^	49.20 ± 2.16 ^d^		
	20	−151.11 ± 4.38 ^e^	72.73 ± 2.11 ^b^	138.18 ± 4.01 ^c^	50.56 ± 1.47 ^c^		
Sodium alginate	5	−100.27 ± 5.31 ^e^	93.65 ± 4.96 ^a^	127.18 ± 6.74 ^cd^	19.24 ± 1.02 ^e^	143.6	0.9659
	10	−219.84 ± 7.91 ^b^	90.59 ± 3.26 ^a^	135.31 ± 4.87 ^bc^	25.88 ± 0.93 ^f^		
	20	−150.04 ± 3.15 ^e^	86.76 ± 1.82 ^a^	149.08 ± 3.13 ^abc^	30.93 ± 0.65 ^e^		
Xanthan gum	5	−135.99 ± 6.66 ^c^	48.11 ± 2.36 ^ef^	101.35 ± 4.97 ^f^	55.78 ± 2.73 ^a^	158.9	0.9669
	10	−226.98 ± 7.26 ^b^	53.00 ± 1.70 ^e^	147.18 ± 4.71 ^ab^	55.88 ± 1.79 ^abc^		
	20	−230.80 ± 12.69 ^a^	62.23 ± 3.42 ^c^	149.13 ± 8.20 ^abc^	56.87 ± 3.13 ^b^		
Microcrystalline cellulose	5	−25.45 ± 0.69 ^g^	49.30 ± 1.33 ^def^	112.08 ± 3.03 ^ef^	37.13 ± 1.00 ^d^	175.3	0.9958
	10	−42.80 ± 1.71 ^f^	58.50 ± 2.34 ^cde^	115.50 ± 4.62 ^de^	38.50 ± 1.54 ^e^		
	20	−74.94 ± 2.62 ^g^	67.12 ± 2.35 ^bc^	122.59 ± 4.29 ^d^	42.80 ± 1.50 ^d^		
Soy protein isolate 90%	5	−113.35 ± 5.67 ^d^	54.50 ± 2.73 ^cde^	117.77 ± 5.89 ^de^	51.19 ± 2.56 ^ab^	118.5	0.9656
	10	−98.74 ± 2.37 ^e^	64.26 ± 1.54 ^c^	120.36 ± 2.89 ^cde^	52.98 ± 1.27 ^bcd^		
	20	−179.70 ± 8.09 ^c^	69.13 ± 3.11 ^bc^	145.27 ± 6.54 ^bc^	56.52 ± 2.54 ^b^		
Whey protein concentrate	5	−151.67 ± 4.70 ^b^	46.70 ± 1.45 ^f^	130.51 ± 4.05 ^bcd^	49.81 ± 1.54 ^b^	95.4	0.99
	10	−166.36 ± 9.32 ^c^	61.13 ± 3.42 ^cd^	126.12 ± 7.06 ^cd^	50.08 ± 2.80 ^cd^		
	20	−160.80 ± 4.50 ^de^	72.43 ± 2.03 ^b^	138.85 ± 3.89 ^c^	51.70 ± 1.45 ^bc^		
Food collagen	5	−12.22 ± 0.53 ^h^	73.99 ± 3.18 ^b^	127.13 ± 5.47 ^cd^	35.19 ± 1.51 ^d^	152.7	0.994
	10	−168.67 ± 6.24 ^c^	55.24 ± 2.04 ^de^	147.22 ± 5.45 ^ab^	60.75 ± 2.25 ^a^		
	20	−177.25 ± 3.90 ^cd^	62.72 ± 1.38 ^c^	159.83 ± 3.52 ^ab^	63.06 ± 1.39 ^a^		
Modified starch	5	−10.20 ± 0.49 ^h^	59.50 ± 2.86 ^c^	102.80 ± 4.93 ^f^	32.10 ± 1.54 ^d^	98.6	0.9750
	10	−11.40 ± 0.38 ^g^	63.10 ± 2.08 ^c^	108.50 ± 3.58 ^e^	34.50 ± 1.14 ^e^		
	20	−12.60 ± 0.66 ^h^	67.40 ± 3.50 ^bc^	115.20 ± 5.99 ^d^	37.80 ± 1.97 ^d^		

Normalized enthalpy (J/g), onset temperature, endset temperature, peak width (°C), activation energy (*E*_a_, kJ/mol) calculated by the Kissinger method, and coefficient of determination (R^2^). Values are expressed as mean ± standard deviation of three independent replicates (*n* = 3); coefficients of variation ranged from 2.0% to 5.6%. Different lowercase superscript letters within the same column and heating rate indicate significant differences among coating materials (one-way ANOVA followed by Tukey’s HSD test, *p* < 0.05); for ΔH, comparisons were performed on absolute values. Ea and R^2^ were obtained from a single Kissinger regression per material across the three heating rates and were therefore not subjected to multiple comparisons.

**Table 2:** Post-spray drying viable count and survival rate of *L. plantarum* microcapsules produced with six protein–polysaccharide combinations.

A: Protein	B: Polysaccharide	Viable Count (CFU/g)	Post-Drying Survival (%)
WPC	Gum Arabic	(7.1 ± 0.05) × 10^8 c^	97.76 ± 0.75 ^a^
WPC	OSA Starch	(1.10 ± 0.007) × 10^8 e^	54.49 ± 0.35 ^d^
SPI	Gum Arabic	(2.8 ± 0.09) × 10^9 b^	64.41 ± 2.13 ^c^
SPI	OSA Starch	(9.8 ± 0.45) × 10^9 a^	66.11 ± 3.02 ^c^
Calcium caseinate	OSA Starch	(1.80 ± 0.026) × 10^8 d^	63.93 ± 0.91 ^c^
Calcium caseinate	Gum Arabic	(7.1 ± 0.17) × 10^7 f^	91.81 ± 2.25 ^b^

Values are mean ± SD (*n* = 3). Different superscript letters within the same column indicate significant differences among the six protein–polysaccharide combinations (ANOVA followed by Tukey’s HSD test, *p* < 0.05). Statistical comparisons of viable counts were performed on log_10_-transformed data (log_10_ CFU/g).

**Table 3:** D-optimal mixture design runs, cell viability (%) and log reduction (Δlog N) after thermal treatment at 92 ± 2 °C for 2 min.

Run	Fraction				Viability	Δlog N
	Trehalose	FOS	Glycerol	Inulin	(%)	(log CFU/mL)
Zero time					100 ± 12.101	0.000
1	0.00	0.50	0.50	0.00	14.300 ± 4.041	0.854 ± 0.125
2	0.00	0.00	1.00	0.00	11.700 ± 0.404	0.931 ± 0.015
3	1.00	0.00	0.00	0.00	11.415 ± 2.421	0.947 ± 0.093
4	0.00	1.00	0.00	0.00	9.145 ± 1.624	1.042 ± 0.077
5	0.00	0.50	0.00	0.50	12.606 ± 2.421	0.905 ± 0.084
6	0.50	0.00	0.00	0.50	12.936 ± 2.021	0.894 ± 0.069
7	0.00	0.00	1.00	0.00	14.321 ± 0.808	0.845 ± 0.025
8	1.00	0.00	0.00	0.00	10.604 ± 0.404	0.976 ± 0.017
9	0.25	0.25	0.25	0.25	11.426 ± 0.808	0.943 ± 0.031
10	0.50	0.50	0.00	0.00	11.100 ± 0.404	0.953 ± 0.016
11	0.13	0.13	0.13	0.63	11.784 ± 2.02	0.935 ± 0.075
12	0.00	0.00	0.00	1.00	8.294 ± 0.404	1.082 ± 0.021
13	0.00	0.00	0.00	1.00	8.001 ± 1.622	1.101 ± 0.088
14	0.50	0.00	0.50	0.00	13.457 ± 0.404	0.872 ± 0.013
15	0.00	1.00	0.00	0.00	9.141 ± 0.808	1.040 ± 0.038
16	0.13	0.13	0.63	0.13	12.625 ± 1.625	0.902 ± 0.056
17	0.00	0.00	0.50	0.50	9.142 ± 0.808	1.040 ± 0.038
18	0.13	0.63	0.13	0.13	5.432 ± 2.021	1.281 ± 0.166
19	0.50	0.50	0.00	0.00	11.727 ± 0.404	0.931 ± 0.015
20	0.63	0.13	0.13	0.13	13.147 ± 1.620	0.883 ± 0.054

Component fractions (X_i_) represent proportions of total thermoprotectant concentration (2.5% *w*/*v*). Zero-time control represents viability prior to thermal challenge (*n* = 3, mean ± SD). No multiple-comparison letters are reported because the runs are design points of a D-optimal mixture design rather than independent treatment groups; differences between compositions are evaluated through the ANOVA of the fitted special cubic Scheffé model (Section 3.3), and replicated design points were used to estimate pure error. Values are expressed as mean ± standard deviation of three independent replicates (*n* = 3). Viability was calculated as S = (N/N_0_) × 100 and the log reduction as Δlog N = log N_0_ − log N, where N is the viable count of the treated sample and N_0_ that of the untreated zero-time control.

**Table 4:** Physicochemical and microbiological characterization of optimized *Lactiplantibacillus plantarum* microcapsules produced by spray drying using a WPC–gum arabic wall system, with and without the optimized thermoprotectant formulation.

Parameter	Microcapsules Without Thermoprotectant	Microcapsules with Thermoprotectant
Viable count post-drying (CFU/g)	(8.5 ± 0.55) × 10^8 b^	(2.4 ± 0.13) × 10^11 a^
Viable count post-thermal 92 °C/2 min (CFU/g)	(2.1 ± 0.19) × 10^6 b^	(6.9 ± 0.54) × 10^9 a^
Thermal resistance at 92 °C/2 min (%)	0.25 ± 0.03 ^b^	2.86 ± 0.27 ^a^
Log reduction after thermal treatment (Δlog CFU/g)	2.60 ± 0.05 ^a^	1.54 ± 0.04 ^b^
Post-drying survival (%)	58.42 ± 2.15 ^b^	73.16 ± 0.64 ^a^
Particle size D(4,3) (µm)	12.18 ± 1.84 ^a^	10.43 ± 2.02 ^a^
Moisture content (%)	8.12 ± 0.81 ^b^	9.49 ± 0.25 ^a^
Water activity (aᵥᵥ)	0.19 ± 0.02 ^a^	0.23 ± 0.02 ^a^
Carr Index (%)	24.3 ± 2.8 ^a^	18.5 ± 2.1 ^b^

Values are expressed as mean ± standard deviation of three independent replicates (*n* = 3); different superscript letters within a row indicate statistically significant differences between formulations (*p* < 0.05).

## Data Availability

The data supporting the findings of this study are available from the corresponding author upon reasonable request.

## Supplementary Materials

The following supporting information can be downloaded at: https://www.mdpi.com/article/10.3390/foods15203749/s1, Table S1: Analysis of variance (ANOVA) for the 3 × 2 factorial design evaluating protein–polysaccharide combinations for *L. plantarum* microencapsulation; Table S2: Analysis of variance (ANOVA) for the special cubic Scheffé polynomial model fitted to the D-optimal mixture design; Figure S1: Residual diagnostics of the special cubic Scheffé model fitted to the D-optimal mixture design (*n* = 20). (a) Normal probability plot of externally studentized residuals; (b) externally studentized residuals versus predicted post-thermal viability (%). Red lines indicate the Bonferroni-corrected outlier detection limits (±5.60); no observation exceeded these limits. The stepped pattern in (a) arises from replicated design points with near-identical responses.
