# Supplementary material for: Thermophysically Guided Design of Spray-Dried Lactiplantibacillus plantarum Microcapsules with Improved Thermal Stability
Source: Foods. 2026 Oct 21;15(20):3749. doi: 10.3390/foods15203749 (PMC13704569; doi:10.3390/foods15203749)
Supplement: Supplementary file 1 [file foods-15-03749-s001.zip › foods-4539195-supplementary.pdf]

Table S1. Analysis of variance (ANOVA) for the 3×2 factorial design evaluating protein–polysaccharide combinations for L. plantarum microencapsulation.

Source	p-value (log CFU/g)	p-value (Viability %)
Model	0.0019	< 0.0001
A-Protein	0.0226	0.0001
B-Carbohydrates	0.0018	< 0.0001
AB	0.0023	< 0.0001
R^2	0.9325	0.9933
Adjusted R^2	0.8763	0.9878
Predicted R^2	0.7300	0.9734

Table S2. Analysis of variance (ANOVA) for the special cubic Scheffé polynomial model fitted to the D-optimal mixture design.

**Source**	**p-value**
Model	0.0092
[(1)]Linear Mixture	0.0071
AB	0.2266
AC	0.2698
AD	0.0412
BC	0.0310
BD	0.0190
CD	0.2725
ABC	0.0173
ACD	0.0021
BCD	0.0219
Lack of Fit	0.1620
R^2	0.9193
Adjusted R^2	0.781
Predicted R^2	0,234

A = Trehalose, B = FOS, C = Glycerin, D = Inulin. Model validated by non-significant Lack of Fit (p = 0.162), indicating adequate fit to experimental data. The difference between R^2 and Adjusted R^2 (Δ = 0.138) is attributed to the inclusion of higher-order interaction terms required to capture synergistic and antagonistic effects among thermoprotectant components.

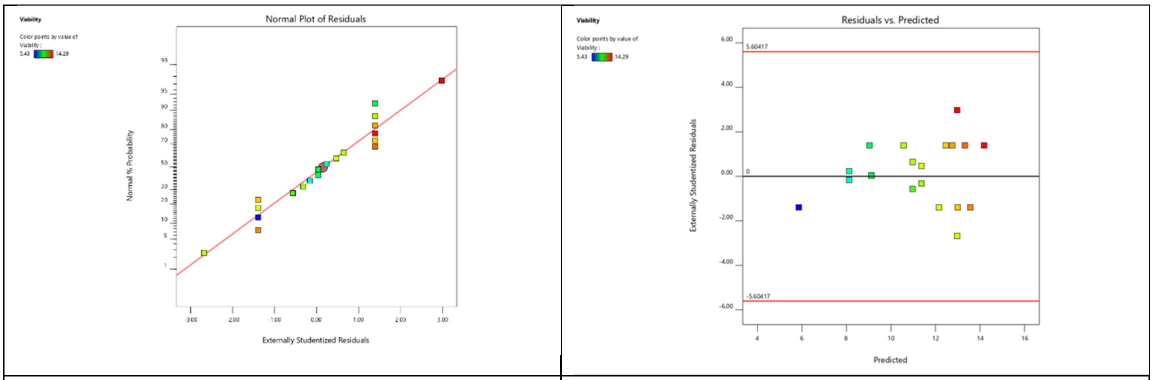

(a) (b)

Figure S1. Residual diagnostics of the special cubic Scheffé model fitted to the D-optimal mixture design (n = 20). (a) Normal probability plot of externally studentized residuals; (b) externally studentized residuals versus predicted post-thermal viability (%). Red lines indicate the Bonferroni-corrected outlier detection limits (±5.60); no observation exceeded these limits. The stepped pattern in (a) arises from replicated design points with near-identical responses.
